# Comment on AlAsmari et al. Venetoclax Induces Cardiotoxicity through Modulation of Oxidative-Stress-Mediated Cardiac Inflammation and Apoptosis via NF-κB and BCL-2 Pathway. *Int. J. Mol. Sci.* 2022, *23*, 6260

**DOI:** 10.3390/ijms27146159

**Published:** 2026-07-10

**Authors:** Bruce A. Trela, Magali R. Guffroy

**Affiliations:** AbbVie, Inc., 1 North Waukegan Rd., North Chicago, IL 60064, USA; magali.guffroy@abbvie.com

We are writing this letter to express our significant concerns regarding the experimental design, experimental data, and interpretation of results in a recent publication by AlAsmari et al. [1].

In that publication, the authors hypothesize that venetoclax (VTX) causes cardiotoxicity through Bcl-2 inhibitor-mediated apoptosis. Following oral administration of venetoclax to rats, the authors describe drug-induced cardiotoxicity characterized by histopathologic changes, increases in cardiac enzymes, and alterations in cardiac hypertrophic gene markers. As detailed below, based on the lack of methodological information and scientific rigor, we consider that the authors should address these deficiencies and remedy accordingly.

Multiple aspects of the manuscript raise significant concerns.

Firstly, two statements in the Introduction section are not accurate or are not supported by the cited references:

- “In the clinical sitting, it has been reported that VTX caused cardiomyopathy and cardiac arrhythmia.” The cited references do not support the contention that VTX causes cardiomyopathy and cardiac arrhythmia. In addition, preclinical and clinical trial data suggest no QTc prolongation and/or cardiac dysfunction related to venetoclax administration.
- “…it has been reported that inhibition of Bcl-2 can lead to apoptosis in different organs, especially the heart, and consequently resulting in organ toxicity.” While Reference 23 describes the well-known hematological toxicities in preclinical or clinical studies with venetoclax and other Bcl-2 inhibitors, References 21 and 22 do not include any information regarding Bcl-2 inhibition and apoptosis in different organs or specifically heart tissue. Reference 21 describes the renal toxicity associated with the rapid release of the intracellular contents of dying cancer cells into the bloodstream (i.e., tumor lysis syndrome) following venetoclax administration to patients with chronic lymphocytic leukemia (CLL). Reference 22 describes the shift toward pro-apoptotic Bcl-2 proteins in various cardiac pathologies but does not specifically provide an example of Bcl-2 inhibitor-mediated apoptosis in the heart.

Secondly, the Materials and Methods section lacks critical information necessary to reconstruct the study. For example, the source of venetoclax and the formulation preparation procedure (concentrations, frequency of preparation, stability) are not provided and plasma concentrations were not evaluated. Given that venetoclax is a ‘Beyond Rule of 5′ compound, with high molecular weight (MW 868), low aqueous solubility (<4.2 ng/mL at pH 4–7.4), and low cellular permeability (Caco-2 Papp 2.3 × 10^−6^ cm/s), an understanding of the formulation preparation and corresponding plasma concentrations are required for interpretation of the data (e.g., extent of absorption, dose–response relationship). Likewise, the histopathology evaluation did not follow standard practices [2]. The evaluation was based on 10 randomly selected 400× fields instead of a comprehensive evaluation of the entire heart sections. It is unclear how the “cardiomyocyte cross-sectional size” was evaluated to provide numerical data since the Materials and Methods section only indicates that the “size was estimated”.

In general terms, this publication does not provide any conclusive evidence for venetoclax-associated cardiotoxicity in rats dosed orally up to 100 mg/kg/day for 21 days. The following comments relate to clinical pathology and/or anatomic pathology endpoints and highlight interpretation inaccuracies.

- There is no significant effect on cardiac troponin I. Creatine kinase (CK)-MB is not a specific biomarker of cardiac injury as it is also present in skeletal muscles and other tissues. In the absence of a significant troponin I increase, the minimal CK-MB increases reported in venetoclax-treated rats are unlikely to be related to cardiac damage. These changes would certainly not qualify as “markedly elevated cardiac enzymes” as concluded in the Abstract of the manuscript.
- There is no effect on absolute heart weight. The minimal increase in relative heart weights is likely related to the slight reduction in body weight in venetoclax-dosed rats.
- The photomicrographs provided to illustrate the histopathological changes do not show evidence of venetoclax-related heart microscopic findings. The slight cardiomyocyte “nuclear enlargement” (Figure 3E) represents normal nuclear size variation (in combination with the plane of sectioning). The minimal “mononuclear cell infiltrate” (Figure 3C) is consistent with a very early stage of “spontaneous progressive cardiomyopathy” that is commonly reported in rats [3,4]. The focal “myxoid degeneration” (Figure 3F) is related to a fixation/processing artifact; it is not a lesion and there is no associated myocardial damage. None of these findings is indicative of venetoclax-associated myocardial injury and, based on the photomicrographs provided, characterizing these as “major changes in histopathology” as stated in the abstract of the manuscript is a misinterpretation.

To support our divergent interpretation of these experimental data, it is noteworthy that there was no evidence of cardiotoxicity in any of the repeat-dose toxicology studies conducted with venetoclax in support of regulatory marketing applications. In pivotal Good Laboratory Practice (GLP) repeat-dose oral toxicology studies ranging in duration from 4 weeks to 9 months with optimized dosing formulations in multiple species (mouse, rat, dog) with robust sample sizes (up to 10/sex/group for rat), there were no venetoclax-related increases in heart weight and no venetoclax-related macroscopic or histopathologic changes indicative of cardiotoxicity. The histopathology evaluations were peer-reviewed and dosages in the GLP studies were comparable to or higher than those cited in the AlAsmari manuscript; venetoclax plasma exposures at the highest dosages in these studies were generally higher (2- to 12-fold) than those required for efficacy in cancer patients. There was also no evidence of significant cardiovascular changes in safety pharmacology studies conducted with venetoclax. In anesthetized dogs, no effects on QTc, blood pressure, or heart rate were observed at the highest doses evaluated. Venetoclax only produced mild reductions in myocardial contractility and cardiac output at the highest dose evaluated, but these changes in cardiac function were not considered clinically relevant due to their minimal magnitude and the lack of concurrent effects on other hemodynamic parameters. In conscious telemetry dogs, venetoclax did not produce any cardiovascular effects up to and including the highest oral dose of 150 mg/kg. The highest doses in safety pharmacology studies resulted in venetoclax plasma concentrations higher than those achieved in humans (6- to 17-fold).

Another peculiar finding from the publication is the reported increase in interferon-γ. Interferon -γ is not thought to be produced by cardiomyocytes [5]. The substantial upregulation of IFN-γ in the absence of infiltrating leukocytes is rather difficult to reconcile.

In summary, the AlAsmari publication contains significant gaps and raises concerns about inaccurate statements regarding venetoclax cardiotoxicity. Consequently, we consider that the authors should address these deficiencies and remedy them accordingly.
